# Associative learning and recollection of olfactory memory during the respiratory cycle in mammals: how is the self cognized in consciousness?

**DOI:** 10.3389/fnins.2024.1513396

**Published:** 2025-01-17

**Authors:** Kensaku Mori, Hitoshi Sakano

**Affiliations:** ^1^RIKEN Center for Brain Science, Wako, Japan; ^2^Department of Brain Function, School of Medical Sciences, University of Fukui, Fukui, Japan

**Keywords:** associative learning, olfactory system, recollection of memory scene, cognition of self, respiratory cycle, decision making

## Abstract

When we are awake and relaxed, various memory-scenes come up in our mind by spontaneous activation of memory engrams. We find ourselves in the memory-scene longing for it by the present self. The memory scene is also recollected by sensory inputs from the surrounding world for learned behavioral decisions. It is well experienced that odorants act as strong cues in remembering associated memory. Associative learning of odor signals and object cognition enables us to predict cognitive imagery of an environmental object. Here, we discuss the neural network connecting the olfactory cortices to the higher cognitive areas that dynamically switches the processing mode from feedforward to top-down. These processes are correlated with the respiratory cycle to form and recollect odor-object associative memory. We infer that during the inhalation phase, feedforward odor signals drive burst firings of a specific subset of pyramidal cells in the olfactory cortex. In contrast, during the subsequent late-exhalation phase, top-down cognitive scene-signals from the higher areas activate again the same pyramidal cells as those activated by the feedforward signals. Reactivation of pyramidal cells during the exhalation phase may induce plastic changes in the inter-areal synaptic connections in the neural network to form associative-learning memory. In this perspective article, we will discuss associative learning and cognition of self in the mammalian olfactory system.

## Introduction

“What are we? Where do we come from? Where are we going?” As Paul Gaugin asked, these questions are commonly raised when consciousness is established and the self is cognized in humans. When we are awake, the presence of ourselves is recognized by sensing the environmental situation and recollecting the associated memory scene with sensory cues. How does our brain generate the image of self in the surrounding world? How does the brain differentiate the self from other individuals? Olfactory, visual, and auditory systems detect and process the information of remote objects in the external world. The somatosensory system detects physical interactions of our body with nearby objects by touching. Visceral, pain, thermo-sensory, gustatory, and retronasal olfactory systems detect the physiological state inside of our body. Cognition of ourselves in the surrounding situation both inside and outside of our body makes us realize the presence of self.

Among the five sensory systems, the olfactory system is particularly sensitive in recollecting the past memory-scene using odors as remembrance cues (Wilson and East, 2021). The present self recognizes the past self in the recollected memory-scene. The self is also cognized by internal information of our body. We (first-person) evaluate ourselves (third person) in the current situation as an object. Self-cognition is made clearer by recognizing other individuals nearby. In mammals, self-consciousness plays an important role in establishing the sociality for the survival of individuals and species. When the self and non-self are recognized, the animal either cooperates or competes with other individuals in getting foods, avoiding dangers, and finding mating partners. After cognizing the self, how does the animal interact with other individuals? Male aggressive behaviors and male–female attractive responses are well studied at the neural circuit levels (Chou et al., 2016). Basic social behaviors are innately programmed without learning from the previous experience. Although the neural circuits that induce instinctive behaviors are hard-wired, innate decisions can be altered by imprinted memory of sensory stimuli during the critical period, which is important for smooth social interactions later in life. If odor inputs are blocked in neonates, the animal demonstrates improper social responses, e.g., avoiding interactions with unfamiliar mice (Inoue et al., 2021).

In the mouse olfactory system, odor signals are sorted into basic qualities and separately distributed to distinct functional domains in the olfactory bulb (OB) during the primary projection of olfactory sensory neurons (OSNs). For instinctive decisions, odor information is further transmitted by the projection neuron, mitral cells (MCs), directly to specific valence regions in the amygdala (Miyamichi et al., 2011; Root et al., 2014; Inokuchi et al., 2017). In addition to the direct pathway for innate olfactory decisions, odor signals are separately processed by the multi-synaptic pathway for memory-based learned decisions (Mori and Sakano, 2021). Binding signals of odorants detected in the olfactory epithelium (OE) are converted to combinatorial patterns of activated glomeruli (Malnic et al., 1999; Mori, 2014), that are transmitted to the anterior olfactory nucleus (AON) by another type of projection neuron, tufted cells (TCs). Odor maps are utilized as search tags to recollect the associated scene in the previous odor experience (Johnson et al., 2005; Mori et al., 2006; Mori and Sakano, 2011). Once the memory scene is recollected, then the valence circuit that used to be linked to the memory engram is reactivated. Thus, the odor quality is imposed on the object based on olfactory memory. In such a situation, the present self cognizes the past self in the memory scene.

How is it then that odor-associated memory is generated and recollected for learned decisions? In this perspective article, we will discuss self-consciousness and formation/recollection of the learned memory scene, summarizing the recent progress in olfactory research.

## Integration of the lateral and medial olfactory signals during respiration

When we are awake and analyzing ourselves in the current situation, we recognize that the surrounding world is always changing. We make an effort to adapt to those changes and seek the strategy of performing intentional behaviors. How does the brain generate the cognition of self in the consciousness? How is the brain motivated to respond to the changes both inside and outside of our body? These questions have long been asked not only by neuroscientists but also by psychologists and philosophers.

**Figure 1:**
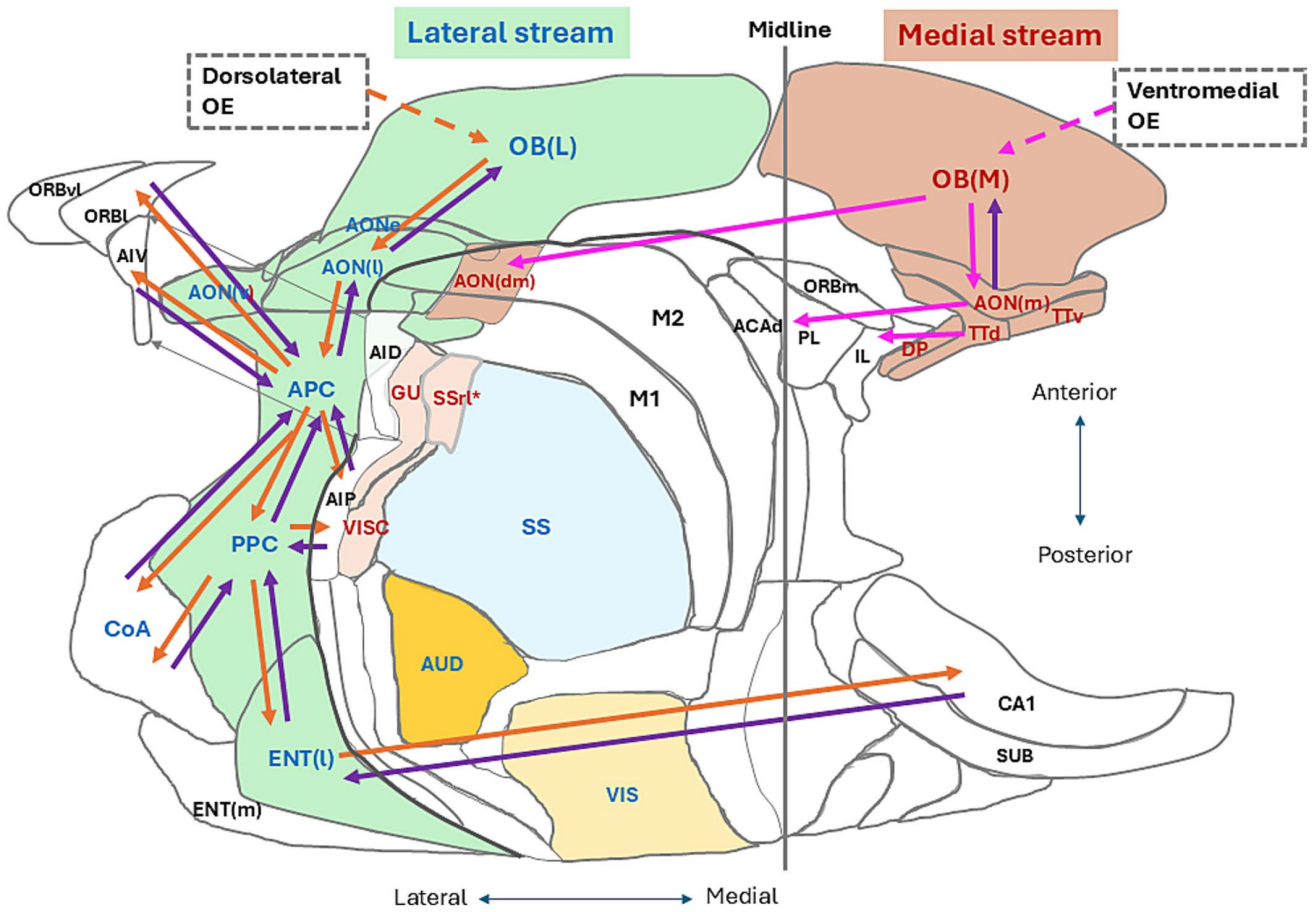
Two olfactory signaling streams in an unfolded map of the mouse cerebral cortex. A dorsal-centered view is shown for the left hemisphere. We propose that the lateral olfactory signaling stream (pale green areas) originates from the lateral map of the olfactory bulb [OB(L)] that receives olfactory sensory inputs from the dorsolateral parts of the olfactory epithelium (OE). We assume that the lateral stream flows to the anterior olfactory nucleus pars externa (AONe), lateral part of the anterior olfactory nucleus [AON(l)], anterior piriform cortex (APC), posterior piriform cortex (PPC), cortical nucleus of amygdala (CoA), and lateral entorhinal cortex [ENT(l)]. Orange arrows in the lateral stream indicate feedforward transmission of orthonasal/external odor signals from the OB(L) to the lateral olfactory cortex (OC) areas and further to the higher multisensory cognitive areas. Purple arrows indicate the top-down flow of cognitive scene signals from the higher cognitive areas. We propose that the medial olfactory signaling stream originates from the medial map of the olfactory bulb [OB (M)] that receives olfactory sensory inputs from the ventromedial part of the OE. We assume that the medial stream flows to the medial part of the anterior olfactory nucleus [AON (m)], dorsomedial part of the AON [AON (dm)], ventral tenia tecta (TTv), dorsal tenia tecta (TTd), and dorsal peduncular cortex (DP). In the medial stream, pink arrows indicate feedforward transmission of retronasal/internal odor signals to the medial OC areas and higher cognitive areas, while a purple arrow indicates the top-down flow from the AON(m) to the OB (M). The higher multisensory cognitive areas include the cortical nucleus of amygdala (CoA), orbitofrontal cortex [lateral orbitofrontal cortex (ORBl), ventrolateral orbitofrontal cortex (ORBvl), and medial orbitofrontal cortex (ORBm)], agranular insular cortex [ventral agranular insular cortex (AIV) and posterior agranular insular cortex (AIP)], medial prefrontal cortex [infralimbic cortex (IL), prelimbic cortex (PL), and dorsal part of anterior cingulate area (ACAd)]. Sensory cortices in the neocortex include the visual cortex (VIS), auditory cortex (AUD), somatosensory cortex (SS), gustatory cortex (GU), and visceral cortex (VISC). Blue letters indicate the sensory areas that process the environmental information, while magenta letters show the areas that process the inside-body information. The rostrolateral part of somatosensory cortex (SSrt*) may process the internal information and is shown in magenta. Some other abbreviations for the cortical areas are listed in the Glossary page. This unfolded map was generated using the Allen Institute for Brain Science (2004). Adapted from Mori and Sakano (2022a).

Here, we try to address two questions: In what time frame does our brain generate cognition of the current self? In what time frame does our brain generate cognition of the current environmental scene? We previously proposed that the lateral map of the OB together with the lateral areas of the olfactory cortex (OC) forms the lateral signaling stream and processes the orthonasal/exteroceptive odor information (Mori and Sakano, 2022a,b) (Figure 1, areas shown in pale green). We assume that the lateral signaling stream (Figure 1, orange arrows) processes the external odor information during the inhalation phase of respiration and transmits it to the higher multisensory cognitive areas, such as the amygdala, orbitofrontal cortex, agranular insular cortex, medial prefrontal cortex, lateral entorhinal cortex, and hippocampus, to generate cognition of environmental scenes. We also proposed that the medial map of the OB with the medial areas of the OC forms the medial signaling stream and processes the retronasal/interoceptive odor information (Figure 1, areas shown in pale brown). We assume that the medial signaling stream (Figure 1, pink arrows) processes the internal odor information during the exhalation phase of respiration and transmits it to higher cognitive areas, such as the medial prefrontal cortex (mPFC) and orbitofrontal cortex. During the exhalation phase, the higher cognitive areas may integrate the cognitive information of the environmental scene and the self-body state to make behavioral decisions (Mori and Sakano, 2024b). Thus, one respiratory cycle appears to provide the cortical networks with a minimum time unit to generate the olfactory cognition of surrounding situation and inner body.

## How is the consciousness established, and surrounding situation evaluated?

Consciousness is generated by sensory information not only from the outside environment but also from inside the body. Sensory systems quickly detect subtle changes in the surrounding world. When the current situation is satisfactory, we become happy and relaxed. Positive neuromodulators, e.g., serotonin, oxytocin, and dopamine, are released and distributed widely in the body making the physiological and mental conditions positive. If the situation is uncomfortable or dangerous, stress hormones, e.g., cortisol and adrenocorticotropic hormone (ACTH), increase inducing proper actions to improve the situation. Based on the previous valence of the memory scene associated with sensory cues, behavioral and emotional outputs are induced, predicting what is going to happen in the current situation. Various memory scenes also come up in our mind by spontaneous firing of memory engrams when the brain is relaxed and resting. If the attention is induced, the memory scene is recollected and elicits motivation to take appropriate actions.

Sensory systems also detect physical problems in our body, for example causing pain as an alert signal. Thus, internal body information is important to maintain healthy homeostatic conditions. When the physiological situation is negatively drifted from normal, e.g., feeling hungry or thirsty, animals take actions to improve the situation. These responses are mostly instinctive and innately programmed without recollecting associated memory. Once the normal body condition is recovered, the desire disappears. In humans, the desire is often motivated by the reward and there is a never-ending goal without achieving satisfaction. This intended desire causes various problems when the reward-motivated behavior dominates over the instinctive feedback regulation that keeps the homeostatic condition normal.

Cognizing the situation both inside and outside of our body makes us realize that consciousness belongs to ourselves, but not to other individuals. How does the brain generate the image of self in the surrounding world? How does the brain differentiate the self and non-self? Figure 1 illustrates the localization of sensory areas in an unfolded map of the left cerebral cortex in mice. The visual and auditory cortices (VIS and AUD in Figure 1) process the information of distant objects in the external world, generating visual and auditory images of the object. We previously hypothesized that the lateral OB map (OB(L) in Figure 1) and lateral regions of the OC process the orthonasal/external odor information, generating the olfactory image of the external world (Mori and Sakano, 2022a,b). Integration of the visual, auditory, and orthonasal olfactory images in the higher cognitive areas may provide the foundation of conscious cognition of the surrounding world.

In contrast, the visceral and gustatory cortices (VISC and GU in Figure 1) in the insula are parts of interoceptive sensory areas that process the physiological information of self-body (Craig, 2015). These cortices form the visceral image of self-body and the taste image of self-mouth. We previously proposed that the medial OB map (OB (M) in Figure 1) and medial regions of the OC are interoceptive sensory areas that process the information of retronasal/internal odors originating from the inside body (Mori and Sakano, 2022a,b) and generate olfactory images of the oral cavity, pharynx, and lung of self-body (Shepherd, 2017). Integration of visceral, gustatory, and retronasal olfactory images in the higher cognitive areas may form the foundation of sensing the physiological state of self-body, namely the internal world. In humans, the interoceptive sensation of self-body is thought to play a key role in generating the sense of ourselves as a self-conscious coherent being (Craig, 2015).

The somatosensory cortex (SS in Figure 1) processes the information of external objects that contact the external surface of self-body, forming the tactile and vibratory images of contact. Integration of these images may form the foundation of cognizing the direct contact of self-body with the external object. The most rostro-lateral part of the SS (SSrl*) is located adjacent to the GU and represents the tongue, pharynx, and esophagus, suggesting that the SSrl* processes the information of internalized foods that contact the inner surface of the oral cavity and digestive tract. This part of the SS may generate tactile images of the internalized foods within the body.

As illustrated in Figure 1, the cerebral cortex separately contains sensory areas for the images of the external world, self-body, and contacts between them. This segregated representation suggests that neural circuits for informational processing of external-world cognition may function independently from those of internal-world cognition. Therefore, we speculate that the cerebral cortex may be the place that differentiates two separate cognitions of the self and non-self. Future studies will elucidate the neural-circuit mechanism for generating integrated images of internal and external worlds, and the interactions between them.

## Respiratory phases and odor-object associative learning

**Figure 2:**
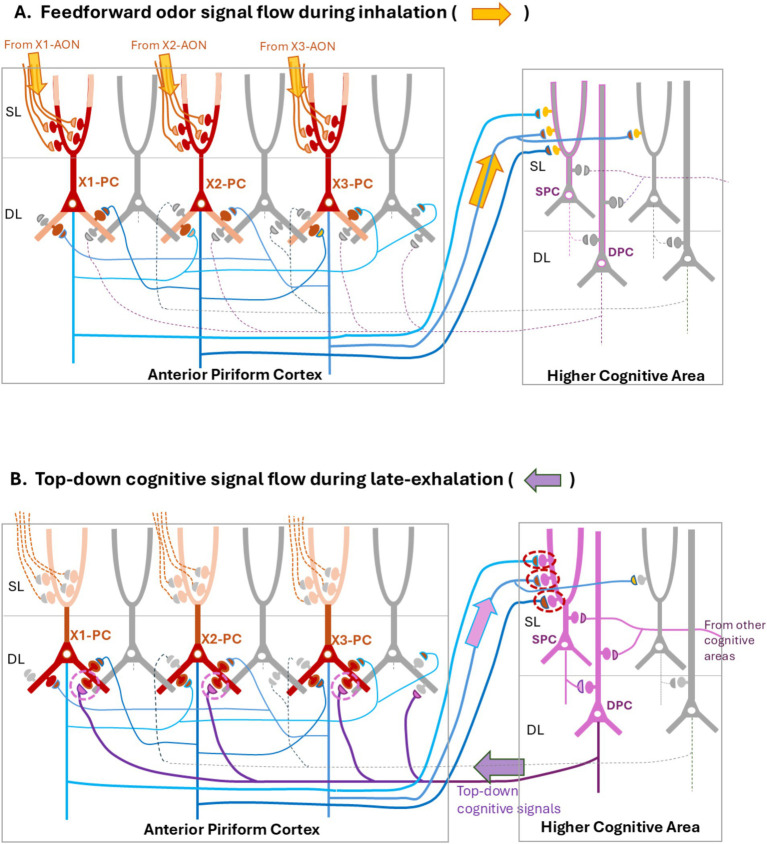
A neural-network model for associative learning of odor signals. We propose that the neural-networks reciprocally connecting the anterior piriform cortex (APC) (encircled left) with the higher cognitive area (encircled right), change the processing mode in relation to the respiratory cycle to generate odor-scene associative memory. Here, we assume that the odor X is composed of three different odorants, X1, X2, and X3. We also assume that odorants X1, X2, and X3 activates APC pyramidal cells (PCs), X1-PC, X2-PC, and X3-PC, respectively. In this model, during the inhalation phase in associative learning **(A)**, feedforward signals from the anterior olfactory nucleus (AON) (orange arrows originated from the X1-, X2-, and X3-responsive AON areas) activate a specific subset of PCs in the APC (X1-, X2-, and X3-PCs, shown in brown). During the late-exhalation phase **(B)**, top-down cognitive scene-signals (indicated by a dark-purple left arrow) from the higher cognitive area re-activate the same subset of PCs in the APC as for the feedforward signals. X-PCs activated by the top-down signals may transmit the signals again to the higher cognitive area (a pale-purple right arrow). Co-activation of X-PCs and cognitive scene-cells may induce plastic changes in the synapses (circled by dashed lines in magenta) connecting X-PCs to the cognitive scene-cells (superficial pyramidal cells, SPC, in purple) after associative learning of odor signals with the scene. This co-activation may also be responsible for inducing plastic changes in the synapses (circled by dashed lines in purple) connecting the cognitive scene-cells (deep pyramidal cells, DPC in purple) to the deep dendrites of X-PCs after the associative learning. After the enhancement of synaptic transmission to X-PCs, spontaneous firing of the cognitive scene-cells may result in the recollection of odor X memory even in the absence of odor inputs. In both figures **(A,B)**, non-activated cells are shown in gray, and their axons are indicated by dashed lines. Thin blue lines in the APC indicate the recurrent axon-collaterals that terminate on the peer PCs. SL, superficial layer; DL, deep layer.

Cognition of an environmental object requires the brain to generate the sensory imagery of the object, i.e., olfactory, visual, auditory, somatosensory, and gustatory imageries. How does the neural circuit in the olfactory cortex generate and store the olfactory imagery? We previously proposed that during the inhalation phase of respiration, the feedforward pathway of the lateral signaling stream, OB(L) → AON(l) → APC → PPC, processes odor information of an object (Narikiyo et al., 2018; Mori and Sakano, 2024a,b) and transmits it to the higher multisensory cognitive areas, such as the amygdala, orbitofrontal cortex, agranular insular cortex, medial prefrontal cortex, lateral entorhinal cortex, and hippocampus (Wang et al., 2020; Ma et al., 2024) (Figure 1). In the APC, feedforward odor signals from the AON(l) to the superficial layer (SL) may drive the APC circuitry during the inhalation phase (Figure 2A). The feedforward transmission of odor signals appears to play a key role in generating the olfactory imagery of environmental objects.

We also proposed that during the subsequent late-exhalation phase, the higher multisensory areas generate cognitive-scene signals of an object for behavioral decision-making and transmit the signals not only to the behavioral output system but also back to the OC areas via top-down pathways (Figure 2B, indicated by dark purple arrow) (Mori and Sakano, 2024b). In the APC, the top-down cognitive scene-signals to the deep layers (DL) may drive the APC circuitry during the late-exhalation phase. Then, what is the functional role of the top-down transmission of cognitive-scene signals during the exhalation phase in generating the olfactory imagery of objects?

Figure 2 shows a neural-network model for associative learning of the odor signals in object cognition. Individual pyramidal cells (PCs) in the APC emit recurrent axon-collaterals that form excitatory synapses with the spines of basal dendrites in the peer PCs (Haberly and Presto, 1986; Johnson et al., 2000; Quraish et al., 2004; Franks et al., 2011; Hagiwara et al., 2012; Yang et al., 2017). It has been proposed that the recurrent collateral-synapses play a key role in learning of the odor input-pattern (Neville and Haberly, 2004; Courtiol and Wilson, 2017; Bolding et al., 2020).

Individual PCs in the AON(l) and APC show the tuning to specific odorant categories (Yoshida and Mori, 2007; Kikuta et al., 2008). Let us assume that the odor quality of an object X is neutral before associative learning, i.e., odor X does not induce any behavioral responses, positive or negative. Let us also assume that the odor of object X is composed of three different odorants (X1, X2, and X3) and that inhalation of these odorants activates X1 pyramidal cells (X1-PCs), X2 pyramidal cells (X2-PCs), and X3 pyramidal cells (X3-PCs), respectively, in the APC (Figure 2A). These PCs in the APC are activated via feedforward pathways consisting of TCs in the OB and PCs in the AON(l) (Mori and Sakano, 2024a,b). Here, inhalation of the odor X causes simultaneous burst firings of all these PCs via the feedforward pathway from the AON (l). Before associative learning, the activity of X-responsive PCs may be transmitted to the superficial layer (SL) of the higher cognitive areas, but the odor signal alone may not induce burst firings of any groups of cognitive scene-cells such as food scene-cells or foot-shock scene-cells in the higher cognitive areas (Figure 2A).

Simultaneous burst firings of the odor-X responsive PCs (X-PCs) may cause mutual excitations via the recurrent axon-collateral excitatory-synapses (Figure 2A, blue axons in the APC), resulting in the relief of ion channels *N*-methyl-D-aspartate receptors (NMDARs) from Mg^++^ blocking in the basal dendrites of PCs. Therefore, burst firings of X-PCs during the inhalation phase may activate NMDARs in the postsynaptic spines (Figure 2A, brown spines in the DL) of their basal dendrites, causing large and long-lasting depolarization that outlasts the inhalation phase.

## Decision making based on the associated memory-scene during exhalation

Emotional empathy plays an important role in establishing smooth social interactions. Social transmission and sharing beneficial information are important for the community in finding foods and avoiding dangers, (Loureiro et al., 2019; Sanchez-Andrade and Kendrick, 2009). If the mice previously experienced foot-shock, they display vicarious freezing simply by watching a cage-mate experiencing foot-shock (Atsak et al., 2011). Let us assume that animals learn the association of odor X with a behaviorally effective stimulus such as foot-shock. This associative learning changes the odor quality of X from neutral to aversive, eliciting the avoidance behavior. We speculate that during associative learning, foot-shock signals activate the foot-shock scene-cells and negative valence-cells in the higher multisensory cognitive-areas (Hegoburu et al., 2014; Biane et al., 2023). In the associative learning of odor X with foods, food signals may activate food scene-cells and positive valence-cells in the higher cognitive areas (Hassan et al., 2023; Biane et al., 2023). After this learning, the neutral odor X becomes attractive and induces positive behavioral responses.

We infer that during the late-exhalation phase for associative learning, a subset of deep pyramidal-cells (DPCs) in the higher cognitive area (Figure 2B, DPC shown in purple) generates cognitive scene-signals for decision making and transmits them back to the cortical sensory areas including the APC. Let us assume that the top-down DPC-axons form the excitatory synapses on the basal dendrites of X-PCs in the APC. Then, we expect that the top-down cognitive scene-signals induce burst discharges of X-PCs, because the firing threshold of these PCs is lowered due to the preceding long-lasting depolarization of their basal dendrites. Only the PCs that have responded to the odor X with burst firings during the inhalation phase may respond to the top-down cognitive scene-signals with burst firings during the subsequent late-exhalation phase. In this way, replay of burst firing of X-PCs during the late exhalation-phase coincides with burst firing of cognitive scene-cells in the higher cognitive areas.

Co-activation of X-PCs and cognitive scene-cells (Figure 2B, SPC) may trigger long-term potentiation of synaptic transmission from X-PCs in the APC onto cognitive scene-cells in the higher cognitive areas (Figure 2B, indicated by dashed magenta circles). Synaptic inputs from X-PCs onto the cognitive scene-cells may activate NMDARs in the postsynaptic spines and cause Ca^++^ influx, triggering long-term synaptic potentiation and enlargement of spines (Figure 2B, magenta circles) (Nicoll and Schulman, 2023; Kasai, 2023), leading to the structural plasticity that potentiates synaptic responses. In contrast, synaptic inputs from X-PCs onto the non-associated non-active cognitive scene-cells (Figure 2B, shown in gray) in the higher cognitive areas may be weakened. Thus, after associative learning, enhanced synaptic inputs from X-PCs alone may be able to selectively activate the associated cognitive scene-cells. For example, after associative learning of the odor X with foot-shock, the animal recollects foot-shock scene-memory based solely on the odor-X inhalation. Formation of associative odor memory of behavioral significance (e.g., attractive foods and aversive danger) is essential for the animal to make appropriate behavioral decisions based on the previous experience. After learning of tight association between the X-PCs and specific scene-cells, both types of cells may form reverberating circuits consisting of the X-PC to SPC connection (Figure 2B, a pale purple arrow), SPCs to DPCs connection (Figure 2, right encircled box), and DPCs to X-PCs connection (Figure 2B, a dark purple arrow).

Another key consequence in the associative learning of odor X with a cognitive scene is that spontaneous recollection of cognitive scene in the higher cognitive areas can induce top-down activation of X-PCs in the APC. As stated above, top-down cognitive scene-signals from the higher cognitive areas reactivate X-PCs during the late-exhalation phase, suggesting that the top-down synaptic transmission from the cognitive scene-cells onto the basal dendrites of X-PCs may activate NMDARs in the postsynaptic spines and trigger the long-term potentiation (Figure 2B, dashed purple circles in the APC). Therefore, we speculate that after generating associative memory of the odor X with a specific cognitive-scene, spontaneous activation of cognitive scene-signals in the higher multisensory cognitive-areas may result in the top-down activation of X-PCs even in the absence of odor X. For example, after the formation of associative memory of banana odor with the visual/tactile cognitive imagery, animals are able to recall the banana odor just by looking at or touching bananas without smelling.

We speculate that the formation of olfactory imagery depends upon not only the odor-induced feedforward activation of PCs in the APC during the inhalation phase but also the subsequent activation of these PCs by top-down cognitive scene-signals during the late exhalation phase. The top-down replay-firing during the late exhalation-phase may explain lingering sensation of the object-odor quality long after the cessation of odor inhalation. After the formation of associative memory of vanilla odor with sugar taste, for example, the vanilla odor has a gustatory quality (Maier et al., 2023). We speculate that during the exhalation phase, top-down cognitive scene-signals are transmitted to all the olfactory, visual, auditory, somatosensory, and gustatory cortices. The cortex of adult mice with rich experiences in the outer and inner worlds may accumulate numerous associative memories of sensory inputs with object/self-cognition. Therefore, we assume that sensory imagery formation of external objects and the self in each sensory cortex depends upon not only the feedforward sensory activation of PCs but also the subsequent reactivation of the same PCs by top-down cognitive scene-signals.

## Self-cognition and social interactions

Mice demonstrate strong curiosity toward unfamiliar mice of both genders. Using ultrasonic vocalization (USV), male mice communicate with females and initiate mounting behaviors. In contrast, male mice demonstrate aggressive behaviors toward male strangers by trying to get rid of them from the territory. It appears that mice are normally shy and avoid stressful interactions with unfamiliar individuals. However, cooperative behaviors in the community are beneficial for finding foods and avoiding dangers. The CA2 region in the mouse hippocampus plays an important role in learning social odor-reward associations and forming episodic social memory (Hassan et al., 2023).

Sensory stimuli during the neonatal period are essential for establishing sociality. In the mouse olfactory system, when the environmental odor inputs are blocked in neonates, mice demonstrate autism-spectrum disorder (ASD)-like behaviors (Inoue et al., 2021). If the occluded naris is reopened a few days before the end of the critical period, no apparent defect is seen in social responses later on. It is well-known that odor perception is affected by olfactory imprinting during the neonatal critical period. Pups exposed to 4-methyl thiazole (4MT) demonstrate attractive responses to 4MT as adults, although its odor quality is innately aversive. A naïve olfactory map whose glomerular arrangement is genetically determined, is further refined and adapted to the olfactory environment after birth in an odor-evoked manner. Plastic changes in the synaptic structure seem to occur in the OB by selectively enlarging the responding glomeruli (Inoue et al., 2018).

Because respiration begins just after birth, the external-odor experience occurs only after the birth. In addition, if respiratory phases play a key role in associating odor-cue signals with a specific cognitive scene, such associative learnings may start in neonates. Therefore, odor exposure during the neonatal period or critical period provides initial opportunity for the odor - cognitive scene associative-learning. Because of the absence or scarceness of pre-formed associative-memory circuits, associative learning during the early life periods may have profound influence on the formation of learned circuits as well as early plasticity of innate circuits.

Olfactory imprinting is triggered by Semaphorin 7A (Sema7A) that promotes the post-synaptic events within the glomeruli (Inoue et al., 2021). Sema7A expression is induced in an odor-evoked manner in OSNs and localization of its receptor Plexin C1 (PlxnC1) in the dendrites of MCs and TCs is restricted to the postnatal days 0 ~ 7. Blocking the Sema7A/PlxnC1 signaling in neonates results in impairment of smooth social interactions as adults. Oxytocin that imposes the positive quality on imprinted memory is also needed for this adaptive learning during the critical period. In the social-memory test, wild-type mice usually lose weariness toward the unfamiliar mice in subsequent encounters. However, the oxytocin KO mice do not become accustomed to the stranger, maintaining an elevated ACTH concentration in the blood stream. It has been shown that oxytocin administration by intraperitoneal injection in the oxytocin KO pups improves the social responses if the oxytocin treatment is given during the critical period (Inoue et al., 2021). It will be interesting to study how the olfactory memory lowers the stress by learning, and how the vigilance gets lowered against unfamiliar individuals in this adaptation.

The sociality also plays crucial roles in constructing the social structure, e.g., finding partners, claiming territory, and establishing the hierarchy in the community. It has been reported that Neuropilin 2 (Nrp2) plays a key role in forming the olfactory circuit for attractive social responses (Inokuchi et al., 2017). During the primary projection of OSNs from the OE to the OB, odor qualities are sorted into aversive and attractive using repulsive axon-guidance molecules, Nrp2 and its repulsive ligand Sema3F (Takeuchi et al., 2010). Aversive information, e.g., predator scents, is transmitted from the OE to the dorsal domain in the OB (Kobayakawa et al., 2007) and then to the negative-valence region in the amygdala (Miyamichi et al., 2011; Root et al., 2014) by the Nrp2^−^MCs. In contrast, attractive social information collected by the posteroventral glomeruli is conveyed by the Nrp2^+^ MCs to the positive-valence region in the amygdala. The Nrp2 KO mice specific for MCs fail to induce the male USV toward female mice, pup suckling, and nursing behaviors. In *in utero* electroporation experiments, ectopic expression of the *Nrp2* gene in the embryonic Nrp2^−^ MCs changes their fate from aversive to attractive, relocating them from the dorsal to the ventral OB and redirecting their projection target to the positive valence region, i.e., anterior medial-amygdala (Inokuchi et al., 2017). It is notable that activation of a single axon-guidance gene, *Nrp2*, is sufficient to change the role of projection neurons from aversive to attractive in the olfactory circuit formation.

## Discussion

In the 17th century, the French philosopher Descartes said “I think, therefore I am.” How is “the self” cognized in our consciousness? It is generally thought that consciousness is generated by sensing the environmental situation in the surrounding world both inside and outside of our body. Behavioral and emotional motivations are elicited by evaluating the situation. For instinctive olfactory responses, the genetically programmed direct pathway makes a decision for output responses without referring the previous experience, i.e., learned memory. These stereotyped responses are the consequence of natural selection during evolution and always biologically correct. In higher vertebrates, behavioral decisions are also made based on the previous experience, recollecting the associated memory-scene with sensory cues. Here, the first-person self is cognized in the consciousness by looking at the third-person self in the memory scene. Thus, the self-cognition appears to play an important role in inducing motivation and desire, making behavioral decisions via the learned circuits. As discussed in this article, it will be important to study further how the sensory input and memory scene are integrated, and how the associated memory-scene is recollected by sensory cues.

After cognizing the self in the recollected memory-scene, we locate ourselves in the cognitive map and determine whether the current situation is satisfactory or not, measuring the distance (difference) between the present self and what it should be. Based on this evaluation of the current self, we take action to achieve the goal or improve the situation. Mice demonstrate good capabilities of physical mapping and goal-positioning in the virtual-reality navigation system (Dombeck et al., 2010; Aronov and Tank, 2014; Chen et al., 2018). We assume that locating ourselves in the cognitive world has evolved from the spatial mapping in the physical world as commonly seen in the animal.

For survival, animals need to attend to both external and internal worlds in searching for food and avoiding danger. Cortical circuits attend to the external information and generate cognitive images of the object with sensory cues. On the other hand, the cortical circuit attends to the internal sensory information of the current physiological state of the self-body, so that the animal can induce or terminate the motivation and desire. How does the cortical circuit allocate the timing of external and internal attentions? Recent progress in our knowledge of the central olfactory system provides us with some hints for answering these questions. As illustrated in Figure 1, the central olfactory system in the cerebral cortex appears to have two distinct signaling streams, lateral and medial. Neural circuits in the lateral stream (Figure 1, orange arrows) are hypothesized to process the orthonasal/exteroceptive odor information during the intentional inhalation phase, generating discrete olfactory images of the external world (Mori and Sakano, 2022a,b). Lateral-stream circuits transmit the external odor information to higher multisensory cognitive areas such as the orbitofrontal cortex, agranular insular cortex, amygdala, lateral entorhinal cortex, and hippocampus. The respiratory inhalation-phase is allocated to the central olfactory circuit and higher cognitive areas to induce the attention of the external odor-world. On the other hand, neural circuits in the medial stream (Figure 1, pink arrows) are assumed to process the retronasal/interoceptive odor information during the exhalation phase to form the current olfactory image of oropharyngeal parts of the self-body. Medial-stream circuits transmit the internal information to the higher cognitive areas in the medial prefrontal cortex including the dorsal-anterior cingulate area, prelimbic cortex, and infralimbic cortex (Bhattarai et al., 2022). Attention to the internal odor-world is called during the intentional exhalation phase. Thus, the external olfactory image appears to be allocated to the lateral-stream circuit and inhalation time frame, whereas the internal olfactory image is allocated to the medial-stream circuit and exhalation time frame, which we call the “one respiratory cycle – one memory scene” hypothesis.

Respiration-phase coherent activity found in the central olfactory areas can widely be seen in other brain regions (Ito et al., 2014; Chi et al., 2016; Biskamp et al., 2017; Zhong et al., 2017; Herrero et al., 2018; Köszeghy et al., 2018; Moberly et al., 2018; Tort et al., 2018; Perl et al., 2019; Bagur et al., 2021; Girin et al., 2021; Kluger and Gross, 2021; Karalis and Sirota, 2022; Folschweiller and Sauer, 2023). In the exploratory behavior, inhalation-phase coherent network-operation occurs not only in the olfactory system, but also in the barrel field of the somatosensory cortex, auditory cortex, and motor cortex. These observations suggest that inhalation-phase coherent operation may take place in the numerous brain regions in processing the sensory information from the external world. In contrast, vocal emission and other emotional expression require the exhalation-phase coherent activity in the cortex. Thus, we extend the above hypothesis to whole cortical networks, speculating that one respiration forms the minimum time frame to generate one behavioral scene leading to one decision-making for the upcoming respiratory cycle.

Voluntary behaviors accompany the intentional respiration. We propose a model of APC-circuitry dynamics in relation to the intentional respiratory cycle, which form the associative learning of an odor signal from the object for behavioral decision-making. Based on this hypothesis, we assume that olfactory image formation in the OC depends upon dynamic interactions between the odor-induced PC activity during inhalation and cognitive-signal-induced PC activity during exhalation. Vocal communication between the self and other individuals is one of the key media for learned social interactions in mammals, which is highly developed as languages in humans. In the vocal communication, it is crucial for the listener’s brain to assess the behavioral significance of received sound-signals in light of the previous experience of sound messages. Thus, it will be interesting to investigate the cortical-network dynamics that generate associative learning of the sensory message-signals for behavioral decisions.

In this perspective article, we focused on the establishment and consolidation of associative learning and suggest that meaningful associative learning occurs over the repeated respiratory cycles involving both inhalation (processing feedforward sensory signals) and exhalation (incorporating top-down cognitive scene signals) phases. However, it should be noted that the feedforward sensory processing takes place very rapidly in a single sniff. Behavioral studies demonstrate that rodents make fine odor discrimination in a single sniff of less than 200 msec (Uchida and Mainen, 2003; Kepecs et al., 2006; Schaefer and Margrie, 2007; Cury and Uchida, 2010), showing the quick and efficient feedforward sensory processing. We speculate that at the early stage of associative learning, the feedforward processing may require longer periods, but once the associative memory is established, feedforward processing may occur rapidly during inhalation. We also speculate that after the establishment of associative memory, the top-down reactivation (scene recollection) may rapidly occur during exhalation. Although the studies of single-sniff recognition demonstrate the remarkable capabilities of the brain in the rapid sensory discrimination, our model highlights how the associative memories and context-dependent interpretations might be formed and replayed during the process that may unfold over repeated inhalation-exhalation cycles.

We postulate that top-down signals from the higher cognitive areas such as the orbitofrontal cortex, agranular insular cortex, amygdala, medial prefrontal cortex, entorhinal cortex, and hippocampus re-activate the pyramidal cells in the olfactory cortex during the late-exhalation phase. However, the precise nature, origin, and timing of the top-down cognitive signals have yet to be studied in detail. We speculate that after the odor - foot shock associative learning (LeDoux, 2000), amygdala networks may link the odor-cue signals with the fear scene and generate the top-down cognitive signals (negative valence) predicting danger. In the case of odor - food associative learning (Rolls, 2006), the orbitofrontal cortex may play a key role in connecting the odor-cue signals with the food scene and generate the top-down cognitive signals that predict the presence of foods. Future analysis of the pyramidal-cell activity in these higher cognitive areas in the awake behaving animal in relation to the animal’s respiration phase may elucidate the basic features of the network dynamics that underly the formation and recollection of associative memory. Future studies are also needed to understand how the neuromodulators such as oxytocin and dopamine interplay with these associative-learning circuits.

We predict that, if our neural-circuit model of odor-object associative learning is correct, optogenetic inhibition of the top-down inputs during exhalation may disrupt associative-memory consolidation, whereas optogenetic augmentation of the top-down inputs may enhance memory formation. We also predict that simultaneous recordings of spike activities or Ca^++^ signals from many pyramidal cells in the olfactory cortex, and that in higher cognitive areas may reveal the feedforward odor-signals locked to inhalation and learned odor - object association-signals locked to exhalation. Our model also suggests that once associative memory is established, top-down cognitive signals alone during exhalation can evoke odor memory. This may predict that blocking of peripheral odor inputs would not prevent the recall of certain odor-associative memory if the memory is already established, although this prediction needs to be verified by future experiments.

Associative memory enables mammals to use the information experienced in the past, so that the animal can predict the future environmental situation and make appropriate behavioral decisions for the survival of individuals and species (Buonomano et al., 2023). However, it remains unclear how the neural networks in the brain determine the timing of forming and using associative memory. We propose that the exhalation phase of intentional respiration provides the temporal dimension for encoding associative memory of olfactory sensory inputs with the cognitive scene. Intentional exhalation may also provide the timing of spontaneous top-down recollection of olfactory associative memory. Inter-areal connections in the cerebral cortex appear to have a common organizational feature consisting of feedforward sensory-signal pathways and top-down cognitive-signal pathways (Barbas, 2015; Vezoli et al., 2021). Future research will clarify whether the intentional exhalation provides the general time frame for all cortical sensory and cognitive systems to form and recollect associative memory. If the spontaneous recollection of sensory memory occurs during exhalation, one respiratory cycle may provide a basic time unit for cognizing the present self in the current surrounding situation, which is influenced by the past sensory experience.

In this perspective article, we have analyzed the neural network activity for associative learning and recollection of sensory memory in relation to the respiratory phases. We have also discussed how the self is cognized in our consciousness and how the motivation is activated. There are two types of motivation. One is the world-driven motivation passively induced by the sensory stimuli, and the other is the self-driven motivation autonomously induced by internal desire. Further studies of these issues at the neural-circuit levels will give new insights into our understanding of the long-standing psychological and philosophical questions: “What are we?” and “Why does such a question arise in our mind?”

## Data Availability

The original contributions presented in the study are included in the article/supplementary material, further inquiries can be directed to the corresponding authors.

## Glossary

ACTH: adrenocorticotropic hormone
AID: dorsal agranular insular cortex
AON: anterior olfactory nucleus
AON(dm): dorsomedial part of the anterior olfactory nucleus
AONe: anterior olfactory nucleus pars externa
AON(l): lateral part of the anterior olfactory nucleus
AON(m): medial part of the anterior olfactory nucleus
AON(v): ventral part of the anterior olfactory nucleus
AUD: auditory cortex
APC: anterior piriform cortex
CA1: field CA1 of the hippocampus
CA2: field CA2 of the hippocampus
CoA: cortical nucleus of amygdala
DP: dorsal peduncular cortex
DPC: deep pyramidal cell
DL: deep layer
ENT(l): lateral entorhinal cortex
ENT(m): medial entorhinal cortex
GU: gustatory cortex
MC: mitral cell
M1: primary motor cortex
M2: secondary motor cortex
mPFC: medial prefrontal cortex
NMDAR: *N*-methyl-D-aspartate receptor
Nrp2: neuropilin 2
OB: olfactory bulb
OB(l): lateral map of the olfactory bulb
OB(m): medial map of the olfactory bulb
OC: olfactory cortex
OE: olfactory epithelium
ORBl: lateral orbitofrontal cortex
ORBm: medial orbitofrontal cortex
ORBvl: ventrolateral orbitofrontal cortex
OSN: olfactory sensory neuron
PC: pyramidal cell
Plexn C1: plexin C1
PPC: posterior piriform cortex
Sema 7A: semaphorin 7A
SL: superficial layer
SPC: superficial pyramidal cell
SS: somatosensory cortex
SSrl*: rostro-lateral part of the somatosensory cortex
SUB: subiculum
TC: tufted cell
TTd: dorsal tenia tecta
TTv: ventral tenia tecta
USV: ultrasonic vocalization
VIS: visual cortex
VISC: visceral cortex
